# P015. Externalizing behaviours in children with headache and epilepsy: a case-control study

**DOI:** 10.1186/1129-2377-16-S1-A144

**Published:** 2015-09-28

**Authors:** Vincenzo Guidetti, Azzurra Antonelli, Sonia Donazzan, Noemi Faedda, Giulia Natalucci, Susanna Simeoni

**Affiliations:** Department of Pediatrics and Child Neuropsychiatry, University “La Sapienza”, Rome, Italy

## Background

Epilepsy and migraine are chronic neurological disorders with episodic manifestations that are commonly treated in neurological practice and frequently occur together [1]. These two disorders share several pathophysiological mechanisms. These mechanisms especially involve neurotransmitter and ion channel dysfunctions [2]. Children with epilepsy or headache are at risk of behavioural disorders that can affect their quality of life. Aim of this study was to analyze the possible correlation between externalizing problems and migraine or epilepsy in children.

## Methods

Four hundred children were tested and divided into three groups (mean age: 10.8±1.73). The experimental group was recruited from the Policlinico Umberto I in Rome, while the control group from elementary and middle schools. The first group included 100 migraineurs divided into migraine with aura, migraine without aura, and tension-type headache. The second was composed of 100 children with epilepsy divided into rolandic, absence, grand-mal, and temporal lobe. The last group had 200 children without any disease. Behavioural problems were screened with the Aggression Questionnaire [3], a standardized and validated instrument.

## Results

Statistical analysis showed relevant differences between the groups. Children with headache had lower scores in all scales compared to the control sample and the epilepsy group (p < 0.05). Children with epilepsy obtained higher scores in physical aggression (p < 0.05) than children without any disease. Moreover, girls, considering the whole sample, had higher scores (p < 0.05) in the hostility scale, while boys had higher scores in physical aggression.

## Conclusions

Results suggest that children with headache tend to inhibit their aggressive behaviours compared to children with epilepsy. On the contrary, children with epilepsy express their anger, hostility, physical and verbal aggression compared to children with headache and without any disease.

Written informed consent to publish was obtained from the patient(s).
